# Thermoregulatory performance and habitat selection of the eastern box turtle (*Terrapene carolina carolina*)

**DOI:** 10.1093/conphys/cox070

**Published:** 2017-12-13

**Authors:** Adam F Parlin, José Pedro S do Amaral, John Kelly Dougherty, M Henry H Stevens, Paul J Schaeffer

**Affiliations:** Department of Biology, Miami University, Oxford, OH 45056, USA; Department of Biology, University of Cincinnati Clermont College, Batavia, OH 45103, USA

**Keywords:** movement, temperature, physiology, thermal ecology, ecophysiology, habitat selection

## Abstract

Environmental conditions may affect individual physiological processes that influence short-term performance and ultimately growth, survival and reproduction. As such, habitats selected by animals must provide suitable and adequate resources. Ectothermic species are highly dependent on climatic conditions and ambient temperatures that dictate body temperature regulation and in turn physiological processes. We investigated the thermoregulatory performance, habitat selection, and movements of an ectothermic vertebrate, the Eastern box turtle (*Terrapene carolina carolina*) to assess the importance of thermoregulatory physiology in habitat selection. We evaluated the relationship between habitat selection and thermoregulatory performance in Southwest Ohio over two active seasons from May until October. We found that *T. carolina* selected shaded habitats, including evergreen and deciduous forests, as well as herbaceous grasslands, conformed to the ambient temperatures throughout the active season, although these habitats had temperatures below those expected based on thermal optima of box turtles. Further, we found that movement was not correlated with internal body temperature. Our study shows that thermal conditions are not paramount in habitat selection of box turtles, but that cooler temperatures do not have an effect on the extent of their locomotion.

## Teaser

Conservation requires knowledge of the interaction between an organism and its environment. Thermal conditions have an important role in habitat selection, movement, and maintenance of physiological processes for ectotherms. We found that box turtles chose cooler temperatures than expected and allowed their body temperatures to match the environment, but that this had little effect on their movement.

## Introduction

Conservation of animals requires understanding of both habitat requirements and the nature of their realized niches (Wikelski and Cooke, 2006). Habitat selection and use by ectotherms depends on the physical characteristics of the habitat, and on the thermal physiology of the species (Díaz, 1997; Lelièvre *et al.*, 2011; Logan *et al.*, 2015). Physical characteristics of a habitat have profound effects on the thermal conditions, generating temperature variation within the habitat and in turn influencing ectotherm physiology (Tracy, 1976; Niehaus *et al.*, 2012). Habitat structure can create thermally heterogeneous conditions with heat sources and sinks (Vitt *et al.*, 2005; Gunderson and Leal, 2012) as well as thermal homogeneity that can limit access to cooler or warmer microclimates (Webb and Shine, 1998). Therefore, data on how habitat structure influences thermal conditions can reveal the suitability of habitats, the degree to which animals can exploit microclimates successfully, and is fundamental for understanding the habitat requirements of ectotherms.

All organisms are constantly exchanging heat with their habitat through conduction, convection, radiation, and evaporation (Tracy, 1976). Many species (including reptiles) use behaviours to maintain body temperatures within a set-point range (*T*_set_), known as the preferred body temperature (Hertz *et al.*, 1993, 1999), at which performance of many physiological processes is at or near optimal (Bonino *et al.*, 2011). Additionally, many species regulate physiological processes, such as heart rate (e.g. Grigg and Seebacher, 1999) to compensate for sub-optimal body temperatures (Seebacher and Shine, 2004; Seebacher and Franklin, 2005). The ability of some reptile species to regulate their body temperature in unfavourable environmental conditions allows the maintenance of prolonged activity (Herczeg *et al.*, 2003; Randriamahazo and Mori, 2004). Active thermoregulators maintain body temperatures by exploiting heat sources and sinks to achieve optimal temperatures for activity, thus extending the range of suitable environmental temperatures (Christian and Weavers, 1996; Grigg and Seebacher, 1999). As an alternative strategy, strict thermoconformers rely on passive heat exchange with the environment, potentially reducing the amount of time and energy spent locating heat sources and sinks; however, thermoconformers depend on the suitability of environmental temperatures (see review Angilletta, 2009).

Thermal variation and habitat structure can make it difficult to maintain body temperatures that are physiologically optimal, and thus can directly impact population persistence, recruitment, and dispersal (Huey and Slatkin, 1976; Huey, 1991). Further, thermally challenging environments can inhibit daily and annual activity patterns (Tuttle and Gregory, 2012), and consistent sub-optimal conditions are likely to reduce population viability. The task of locating suitable habitat requires that individuals are able to orient themselves throughout the area they occupy and to navigate to new locations (Able, 1991; Benhamou, 2004). Of increasing concern is how habitat fragmentation will impact species persistence as fragmented habitats tend to be hotter, drier, and more variable in their micro-climate conditions (Tuff *et al.*, 2016). Therefore, fragmented habitats can be a challenge to persistence due to the physical characteristics of the patches, or if distance between habitat patches challenges the capacity for dispersal (Hokit and Branch, 2003; Iglay *et al.*, 2007; Watling *et al.*, 2011; Wittenberg and Beaupre, 2014). Changing climate conditions will further impact habitat quality (Kearney, 2013), dispersal (Buckley *et al.*, 2013), and ultimately distribution (Kearney and Porter, 2009) of many species.

Studies of the thermal physiology of free-living animals provide understanding of how thermoregulatory challenges influence animal–habitat interactions as well as movement ecology, and potentially inform management decisions based on understanding of habitat quality grounded in animal function. Recently, major advances in biologging technology have increased our ability to quantify the movements and physiological performance of free-ranging animals at high-temporal and spatial resolution, permitting more detailed studies of animal–habitat interactions (Bograd *et al.*, 2010; Wilson *et al.*, 2015). Our study focused on the movement ecology and field physiology of Eastern box turtles (*Terrapene carolina carolina)*. Box turtles are listed as a species of special concern (IUCN, 2016) and there is limited field collected data on their physiological ecology near the northern edge of their distribution in the Midwestern United States, where much of their habitat is fragmented. Box turtles have been documented as a temperate, terrestrial turtle species with the ability to tolerate freezing (see review Dodd, 2001), thus permitting occupancy of thermally challenging environments with shorter spring and fall seasons and thus limited seasonal activity. Box turtles provide an ideal opportunity to record high-resolution spatial, temporal and physiological data simultaneously in a terrestrial ectotherm because of their exceptional ability to carry external loads (Marvin and Lutterschmidt, 1997; Wren *et al.*, 1998).

To gain insight into these parameters, we investigated the effects of temperature on habitat occupancy and determined how seasonal thermal variation influenced thermoregulatory performance. To further test the relationship between habitat selection and thermoregulatory behaviour, we determined the extent to which thermal habitat influences daily movement. We measured the thermal characteristics of the habitats available to box turtles within the study area to assess the thermal quality of those habitats as related to the preferred temperatures of turtles. We measured organismal thermoregulatory behaviour to assess the realized performance of the turtles as a measure of how thermoregulatory behaviour impacts habitat selection. Finally, we characterized the extent of movement in relation to thermoregulation to assess how thermoregulatory behaviour and habitat choice would impact the ability to forage and disperse. We hypothesized that (1) thermal quality of the habitat selected would match preferred body temperatures, (2) box turtles would rely on thermoconformity given their limited mobility and (3) hourly distance moved would be positively related to body temperature.

## Methods

### Study sites

The study was conducted within Butler County in Southwest Ohio (39.5° N, 84.7° W) in three different forest patches: Hueston Woods State Park (HW), Miami University Natural Areas (MUNA) and the Miami University Ecology Research Center (ERC). We conducted the study from May until October in 2014 and 2015. Forested habitat in Southwest Ohio is extremely fragmented due to the high extent of agricultural fields. The MUNA and HW are mid- to late-successional forests containing primarily sugar maple (*Acer saccharum*), red and white oak (*Quercus rubra* and *Q. alba*), ash (*Fraxinus spp.*), beech (*Fagus grandiflora*), and black walnut (*Juglans nigra*). Planted white pine (*Pinus strobus*) occurs in stands or along roadsides and parking in some areas. The ERC is composed of agricultural fields, prairies, shrubs and small forest fragments (Summerville *et al.*, 2001). Climate in this region is characterized as humid continental: warm to hot summers that are occasionally humid, cold winters that are sometimes severe, and precipitation is distributed throughout the year (Karl and Koss, 1984).

### Animals and study design

We captured male box turtles (average of 451.0 ± 20.1, range of 300–578 g body weight) by hand, and data logging devices (procedure described below) were both attached and implanted to a total of 26 turtles (2014, *n* = 14; 2015, *n* = 12). All turtles had BD-52 radio-transmitters (Holohil, ON, Canada) epoxied to the top of the shell. We did not use females in this study given their conservation status to avoid any population effects due to death or impaired egg production following logger implantation. Data logging devices recorded internal body and external micro-climate temperatures, and GPS locations (2015 only for GPS logging) while turtles were in the field. Each individual turtle was monitored for 12–15 days, as described below, during one of the following periods. All data logging occurred during either the beginning (May–June; 2014, *n* = 5; 2015, *n* = 4), middle (July–August; 2014, *n* = 4; 2015, *n* = 6), or end (September–October; 2014, *n* = 5; 2015, *n* = 2) of the box turtle active season. GPS locations were recorded upon capture and, after attaching devices, turtles were returned to the capture location for monitoring.

### Movement, area occupied and habitat selection

Location data were used to characterize movement, and areas and habitat types used by turtles. In 2014, turtles were located daily and GPS coordinates of turtle location were taken between 08:00 and 10:00 h (to standardize the measure) using a handheld GPS unit (GPSmap 62s, Garmin, KS, USA). In 2015, turtles had GPS coordinates recorded at 1-h intervals from 07:00 to 19:00 using animal-borne Bird 1AA2 GPS-Accelerometer tags (e-obs, Grünwald, Germany). To quantify habitat selection, we first defined the availability of each habitat type during the study. The area for available habitat was based on a radius around the initial capture location for turtles, defined by the furthest linear distance from the initial capture recorded for a turtle during the study (456 m). The characteristics of that area were then determined for each of the turtles. We analysed the habitat occupancy data using compositional analysis as proposed by Aebisher *et al.* (1993) however we found that individual box turtles were separated into three distinct groups based on the habitats occupied. This violates the requirement of this analysis for similar habitat selection among individuals in this analysis. This violation was noted by the authors and also stated in the analysis software package (adehabitatHR; Calenge, 2006). Given that we were primarily interested in a combined population process, following Vignoli *et al.* (2017), we also used the analyses of Neu *et al.* (1974), although these introduce the problem of redundancy in the habitats available for use Aebisher *et al.* (1993). We compared actual use to the assumption that habitat usage was proportional to habitat availability, considering each habitat type separately (Neu *et al.*, 1974) using land-use land-cover (LULC; 30m resolution) data.

To determine further characteristics of current home range (during 2-week monitoring period), we calculated 95% Kernel Density Estimates (KDE) for every turtle using the R package adehabitatHR (Calenge, 2006) to compare canopy cover density (30 m resolution), and slope (1 m resolution). Although we had only one location per day in 2014, these data were included as comparison within 2015 of all data points vs. only the first location of each day yielded nearly identical results. We selected canopy cover because of its influence on radiative, conductive and convective heat fluxes that, in turn, determine the thermal quality of the habitat. Slope was selected because orientation mechanisms proposed for turtles have included local topographic landmarks and geotaxis (Caldwell and Nams, 2006). Slope was calculated from digital elevation model (DEM) data of Butler County (Ohio Geographically Referenced Information Program, 2015) in ArcGIS 10.2. Land-use land-cover data and canopy cover density were obtained from Multi-Resolution Land Characteristics Consortium (MRLC; Fry *et al.*, 2011).

### Thermal habitat classification and operative model temperatures

Habitats in the study areas were first divided into two general categories (open and closed) based on how canopy cover influences the thermal quality of the habitat. Open habitats consisted of tall grass fields with plant height between 0.5 and1.25 m and short mowed grass typically lower than 8 cm. Closed habitats consisted of the dense interior of a mature forest patch, forest edge, patchy juniper woodland, and tree lines separating agricultural fields. Open habitats were more exposed to conditions such as solar radiation, wind, and rain that lead to higher temperature fluctuations. Closed habitats were protected from solar radiation and wind, and were expected to be more thermally stable.

We used randomly placed operative models containing iButtons in open and closed habitats to record the range of temperatures that a box turtle could experience in those habitats. Temperature of the operative models (*T*_e_) represents the temperature of an organism in the absence of thermoregulatory capabilities, such as metabolic heat production or evaporative cooling (Bakken, 1992). Given the morphology of box turtles, we used cylindrical (*r* = 40 mm, *h* = 115 mm), water-filled (450 ml), high density polyethylene bottles that were painted flat brown as models to measure *T*_e_. Teflon tape was wrapped around the bottle threads to prevent models from losing water. Temperatures of the models were nearly identical with those of a deceased turtle body over a 24-h period in both field and lab conditions (*T*_model_ = 15.95 ± 7.44, *T*_corpse_ = 15.96 ± 8.14; *P* > 0.05).

Models were placed during 2014 and 2015 while turtles were being monitored in the field. In 2014, each model (*n*_open_ = 20, *n*_closed_ = 40) recorded *T*_e_ data at 15-min intervals. In 2015, models (*n*_open_ = 15, *n*_closed_ = 15) recorded *T*_e_ data at 10-min intervals. All model placements were based on random GPS coordinates in open and closed habitats at MUNA and ERC in both 2014 and 2015. We omitted from analysis models that were punctured or leaked during monitoring.

### Data logger implantation surgery

Implanted biologging devices recorded internal body temperature (*T*_b_), external shell temperature (*T*_shell_), and heart rate—although heart rate is not reported in this paper. For surgery (in the MU animal care facility) to implant the logging devices, turtles were first placed into a small induction chamber flushed with a 95:5% O_2_:CO_2_ mix for 15 min to induce hyperventilation. Subsequently, isoflurane (5%) was added to the gas mix flowing into the chamber. Upon loss of righting response, which took ~45 min, turtles were restrained and had an endotracheal tube inserted into their epiglottis to continue administering isoflurane (2%) in 100% oxygen via ventilator (Euthanex Corp, PA, USA). Ventilator was set at 10 bpm with a 40% inspiration time, tidal volume of 9.0 ml and a pulse pressure of 10.5 cm H_2_O. Once animals were anesthetized and the incision region sterilized with 70% isopropyl alcohol and Betadine, we implanted a DS1922L iButton (Maxim Integrated, CA, USA) into the left posterior region of the coelomic cavity, anterior to the hind limb to record internal temperature. Size of the incision site was ~2 cm and was sutured using non-absorbable suture and sealed with cyanoacrylate glue. An ECG-tag 1AA2 (e-obs, Grünwald, Germany) was epoxied to the top of the carapace and the electrode leads were inserted through two holes drilled in the carapace (~4.5 mm each in diameter) at the R4 and L8 marginal scutes. To prevent secondary infection, any opening in the carapace after the ECG leads were inserted was filled with sterile bone wax. Electrode leads were coated with epoxy to prevent damage from field conditions and to hold them in place. In addition to recording heart rate, the ECG-logger recorded external (shell) temperature.

Implanted iButton and external ECG-logger recorded *T*_b_ and *T*_shell_ simultaneously at 5-min intervals and no difference between the two were found (*t*_115_ = 1.98, *P* > 0.05). Internal and external data loggers were tested against each other prior to the experiment. Turtles were given a 96-h recovery period before being released back into the field for monitoring. iButtons were programmed to start recording at midnight after a minimum of a 24-h field acclimation after release. At the end of the monitoring period, animals were recaptured and the instruments removed. Note that several animals with fully healed carapaces were recaptured later in the study. Surgery procedure followed approved MU Institutional Animal Care and Use Committee protocol #906. Total weight of the devices was between 7% and 13% of each turtle's total body mass; in 2014 the weight of devices was 35 g and in 2015 it was 55 g.

### Box turtle thermal preference

Preferred body temperature (*T*_set_) of box turtles was determined using previously collected data from do Amaral *et al.* (2002). In that experiment, male box turtles (*n* = 11) were acclimated to a 12:12 L:D photoperiod at 20 ± 1.0°C for 14 days. *T*_set_ was tested using a linear thigmothermal gradient box with temperatures maintained at a range of 6.0 ± 1.5°C–44.0 ± 1.5°C. Box turtle body temperature (*T*_b_) was recorded once every 5 min for 48 h. Thermal preference, or set-point temperature, is traditionally calculated using the central 50% or 80% of body temperatures recorded in an artificial laboratory gradient independent of ecological constraints (Hertz *et al.*, 1993). We first calculated the preferred body temperature range using the central 50% of body temperatures recorded in the laboratory gradient and applied it as a constant (*T*_set-C_) across a general 24-h period. As thermal requirements may vary throughout with a diurnal cycle, we also calculated, with the same laboratory data, a dynamic preferred body temperature (*T*_set-D_) for a general 24-h period by pooling data by hour and using the central 50% of temperatures selected within each hour. Both *T*_set-C_ and *T*_set-D_ ranges were used in calculations of thermoregulatory indices (below) for comparative purposes.

### Indices of thermoregulation

We used the modifications proposed by Blouin-Demers and Weatherhead (2001) to the original thermoregulatory indices of Hertz *et al.* (1993) to quantify thermoregulatory behaviour in this study. We used the following indices: thermal accuracy (*d*_b_), thermal habitat quality (*d*_e_), effectiveness of thermoregulation (*E*′), and micro-climate exploitation (*E*_m_). Thermal accuracy is the deviation of the body temperature (*T*_b_) from the preferred body temperature (*T*_set_) and is calculated as *d*_b_ = │*T*_set_−*T*_b_│. Given that *T*_set_ is a range, values of *T*_b_ within its upper and lower bounds are calculated as zeroes. Thermal habitat quality is the deviation of the operative model temperature (*T*_e_) from the preferred body temperature (*T*_set_), similarly calculated as *d*_e_ = │*T*_set_−*T*_e_│. The effectiveness of thermoregulation, *E*′, is the difference of these indices (*E*′ = *d*_e_−*d*_b_). Positive values for *E*′ indicate thermoregulation, values of zero indicate thermoconformity, and negative values indicate presence in thermally unfavourable habitats. We define *E*_m_ as the percent of time that the turtle achieves its preferred body temperature across the experienced thermal habitats. To calculate this micro-climate exploitation, we used the *T*_shell_ as a measure of micro-climate temperature selected, and report the percentage of time that body temperature was within the *T*_set-D_ and *T*_set-C_ ranges while *T*_shell_ was above, within, or below the *T*_set-D_ and *T*_set-C_ ranges.

### Data analysis

We first determined the thermal preference of the turtles as a constant (*T*_set-C_) or as a dynamic (*T*_set-D_) range using the central 50% of the data recorded in the thermal gradient for the entire day or each hour, respectively. We next determined characteristics of selected habitats using two approaches. Habitat selection was determined with chi-square analysis comparing the degree of use for each habitat by box turtles following methods of Neu *et al.* (1974). We compared canopy cover and slope of turtle home range with the buffer area previously determined. We assumed that canopy cover density and slope were independent of each other, thus we performed separate paired **t**-tests comparing area occupied by turtles to their available habitat for each. To compare thermal quality of the open to the closed (forest) habitat, we compared the percentage of time (as mean hour) that operative models recorded temperatures within turtle preferred body temperatures for open and closed habitats using a **t*-*test.

Thermal data were categorized into three different groups based on the months of monitoring: May and June (beginning), July and August (middle), and September and October (end). To assess thermoregulatory behaviour, we estimated the effects of year and season on operative model temperature (*T*_e_) and thermal quality (*d*_e-C_ and *d*_e-D_) with linear-mixed models using the operative model as a random effect. Next, we estimated the effects of year and season on body temperature (*T*_b_), shell temperature (*T*_shell_), and thermal accuracy (*d*_b-C_ and *d*_b-D_) independently of the thermal environment using linear-mixed models with the turtles as a random effect. Next, we estimated the effects of year and season on effectiveness of thermoregulation (*E*′) with linear mixed models using the turtles as a random effect. Interactions were tested with chi-squared tests of nested models fitted with maximum likelihood. When interactions were significant, unique combinations were considered significant if their bootstrapped 95% confidence limits did not overlap (Bates *et al.*, 2015). When interactions were not significant, main effects were tested using type III SS ANOVA (Fox and Weisberg, 2011). Lastly for thermal data, we determined the percentage of time (as mean hour) that *T*_shell_ was either above, inside, or below the thermal preference (*T*_set-C_, *T*_set-D_) while corresponding *T*_b_ was inside the *T*_set-C_ or *T*_set-D_ range

To determine the effects of temperature and time of day on movement, using a linear model, we regressed distance moved in an hour against the corresponding internal body temperature. Additionally, we used an ANOVA to compare the mean distance moved and time of day during hours of monitoring. Skewed data were corrected with log-transformation where appropriate. In all cases data were analysed in R version 3.0.2 (R Core Team, 2016) and α was set at 0.05.

## Results

### Thermal preference

**Figure 1:**
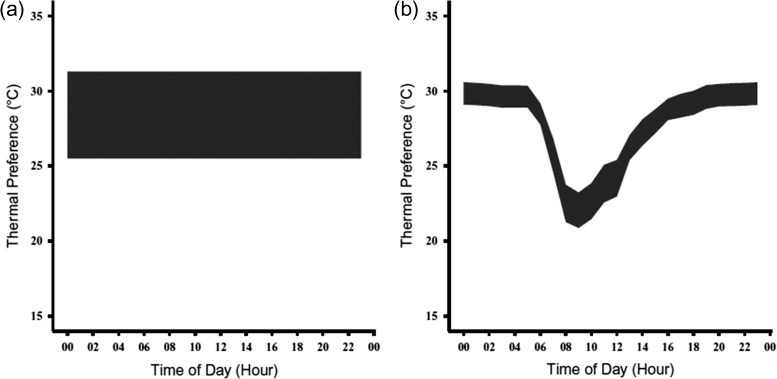
Constant (**a**) and dynamic (**b**) *T*_set_ calculated from data collected by do Amaral *et al.* (2002) presented across a representative day. The central 50% of temperatures selected for the *T*_set-C_ ranged from 25.5°C to 31.3°C. Using this approach, the temperatures selected are considered to unchanged throughout the day. The *T*_set-D_, using the central 50% of selected temperatures for each hour, shows that when given the opportunity, box turtles will select warmer body temperatures at the beginning and end of the day and cooler temperatures in the middle of the day. Additionally, the *T*_set-D_ had a narrower range than *T*_set-C_, between 0.7°C and 1.3°C for each hour.

Preferred body temperatures (*T*_set_) were calculated as a constant (*T*_set-C_) and as a dynamic function of the time of day (*T*_set-D_). Constant preferred body temperature (*T*_set-C_) produced a broad range of selected temperatures from 25.5–31.3°C. The dynamic *T*_set_ (*T*_set-D_) resulted in a narrower range (0.7–1.3°C for each hour), compared to *T*_set-C_ (Fig. 1). The *T*_set-D_ revealed that, when given the opportunity, box turtles selected warmer temperatures at night (between 00:00–06:00 and 19:00–23:00) and cooler temperatures during the day (between 06:00 and 19:00).

### Habitat selection and environmental temperature exploitation

Generally, box turtles selected closed habitat with relatively dense canopy cover. Ten land-use land-cover categories (LULC, see Table 1) were recorded in the available habitat for box turtles (456-m radius buffer around the release point). Chi-square analysis showed that box turtle observations were proportionally higher in deciduous and evergreen forests, and in herbaceous grasslands relative to the available habitat. Observations in open water, developed areas, mixed forest, cultivated croplands, and pasture and hayfields were proportionally lower relative to the potential surrounding habitat (*χ*^2^ = 2031.57, df = 9, *P* < 0.05, Table 1). Compositional analysis gave nearly identical results. There were no significant differences between geographic slope (as percent rise) comparing home range (5.34 ± 0.5%) and available habitat (5.45 ± 0.4%; **t**_21_ = 0.67, *P* > 0.05). Canopy cover density for home range box turtles (61 ± 5 %) was significantly higher than in the potential area available (48 ± 4%; **t**_18_ = −2.34, *P* < 0.05).

**Table 1:** Chi-square analysis of habitat types in the available area for box turtles (*Terrapene carolina carolina*)

Habitat type	Total available area (m^2^)	Proportion of total area (*p_i_*)	Number of turtle observations	Expected number of turtle observations	Proportion observed in each area $\left({\bar{p}}_{i}\right)$	Bonferroni confidence interval
Developed Area^a^	30 211	0.141	94	269	0.049	0.035 ≤ *p*_1_ ≤ 0.063
Open Water^a^	4 044	0.019	0	36	0	0.000 ≤ *p*_2_ ≤ 0.000
Deciduous Forest^b^	101 647	0.475	1034	906	0.542	0.510 ≤ *p*_3_ ≤ 0.575
Evergreen Forest^b^	12 815	0.060	435	114	0.228	0.201 ≤ *p*_4_ ≤ 0.255
Mixed Forest^a^	5 603	0.026	6	50	0.003	0.000 ≤ *p*_5_ ≤ 0.007
Shrub and scrub	1 608	0.007	14	14	0.007	0.002 ≤ *p*_6_ ≤ 0.013
Grassland and Herbaceous^b^	2 387	0.011	141	21	0.074	0.057 ≤ *p*_7_ ≤ 0.091
Pasture and Hay^a^	29 237	0.137	178	261	0.093	0.075 ≤ *p*_8_ ≤ 0.112
Cultivated Crops^a^	26 118	0.122	4	233	0.002	−0.001 ≤ *p*_9_ ≤ 0.005
Emergent Herbaceous Wetlands^a^	146	0.001	0	1	0	0.000 ≤ *p*_10_ ≤ 0.000
Total	213 821		1906	1906		

GPS locations were from turtles monitored in 2014 and 2015. Habitat use assumes proportional use of the total area (*p_i_*). Confidence intervals are around the proportion of turtles observed in each area $\left({\bar{p}}_{i}\right)$. If *p_i_* is above the confidence interval of ${\bar{p}}_{i}$, then the turtle spends less time than expected, indicating avoidance of that habitat (a). If the *p_i_* is below the confidence interval of ${\bar{p}}_{i}$, then the habitat type is preferred (b). Otherwise, habitats were used in proportion to availability.

Although box turtles selected relatively dense canopy cover, forested habitats and open grassland areas, operative models in closed habitats had significantly lower percentages of time in which operative temperatures (*T*_e_) were within *T*_set_ compared to open habitat. For the constant *T*_set_, *T*_e_ were inside the *T*_set_ range for 18.5 ± 1.0% of the time in open habitat, and for 2.4 ± 0.6% of the time in closed habitat (*t*_239_ = −13.64, *P* < 0.05). For the dynamic *T*_set_, *T*_e_ were inside the *T*_set_ range for 8.6 ± 0.5% of the day in open habitat and for 3.1 ± 0.6% in closed (*t*_292_ = −6.51, *P* < 0.05) calculations.

### Seasonal operative temperatures and thermal habitat quality

Operative model temperature and thermal quality of the habitat varied by season and year. Operative temperatures (*T*_e_) from models used to quantify thermal habitat quality in 2014 and 2015 had statistically significant year *x* season effects (*χ*^2 ^= 549.6, df = 2, *P* < 0.05) for all models. The thermal quality (*d*_e_), calculated using both the *T*_set-C_ (*χ*^2 ^= 212.6, df = 2, *P* < 0.05) and *T*_set-D_ (*χ*^2^ = 268.6, df = 2, *P* < 0.05), had similarly significant year and season interaction effects. For *T*_e_, *d*_e-C_ and *d*_e-D_, bootstrapping confidence intervals overlapped only during the beginning and middle of the 2015 active season, but did not overlap for any other season and year combinations. In each season, open habitats had on average 2.1°C higher (9.9% difference) operative temperatures than closed habitats (Table 2). Closed habitats in 2014 and 2015 increased in average *T*_e_ from the beginning to the middle of the active season, and then decreased from the middle to the end. Open habitats in 2014 decreased in average *T*_e_ from the beginning to the end of the active season. However in 2015, average *T*_e_ increased from beginning to middle and then decreased from middle to end of the active season. Thermal habitat quality (*d*_e_) was higher (lower values indicate similarity of *T*_e_ to *T*_set_) in open habitats compared to closed habitats during each part of the active season (Table 2).

**Table 2:** Operative model temperature (*T*_e_) and thermal habitat quality (*d*_e-C_; *d*_e-D_) for open and closed habitats in 2014 (*n* = 60) and 2015 (*n* = 30) across the entire active season

	Season	Habitat	*T*_e_ (°C)	*d*_e-C_ (°C)	*d*_e-D_ (°C)
2014	Beginning	Closed	19.76 ± 0.10	5.94 ± 0.09	7.24 ± 0.11
		Open	22.09 ± 0.31	5.10 ± 0.21	6.52 ± 0.24
	Middle	Closed	20.06 ± 0.10	5.63 ± 0.09	6.90 ± 0.11
		Open	21.92 ± 0.38	5.03 ± 0.25	6.19 ± 0.29
	End	Closed	14.18 ± 0.15	11.45 ± 0.14	12.75 ± 0.14
		Open	14.91 ± 0.36	11.11 ± 0.30	12.38 ± 0.32
2015	Beginning	Closed	20.04 ± 0.23	5.48 ± 0.23	6.83 ± 0.27
		Open	23.46 ± 0.42	4.22 ± 0.25	5.73 ± 0.29
	Middle	Closed	21.29 ± 0.15	4.24 ± 0.15	5.59 ± 0.21
		Open	24.21 ± 0.30	2.80 ± 0.18	4.13 ± 0.23
	End	Closed	18.14 ± 0.25	7.37 ± 0.25	8.69 ± 0.29
		Open	19.26 ± 0.34	6.70 ± 0.28	7.98 ± 0.32

Higher values indicate thermally inadequate habitat and lower values indicate thermally adequate habitat. In 2014 and 2015, closed habitats had lower temperatures than open during the entire active season. Open habitats were generally more thermally adequate than closed habitats. 2015 had more thermally adequate temperatures than in 2014. Data are presented as weighted means ± standard error.

**Figure 2:**
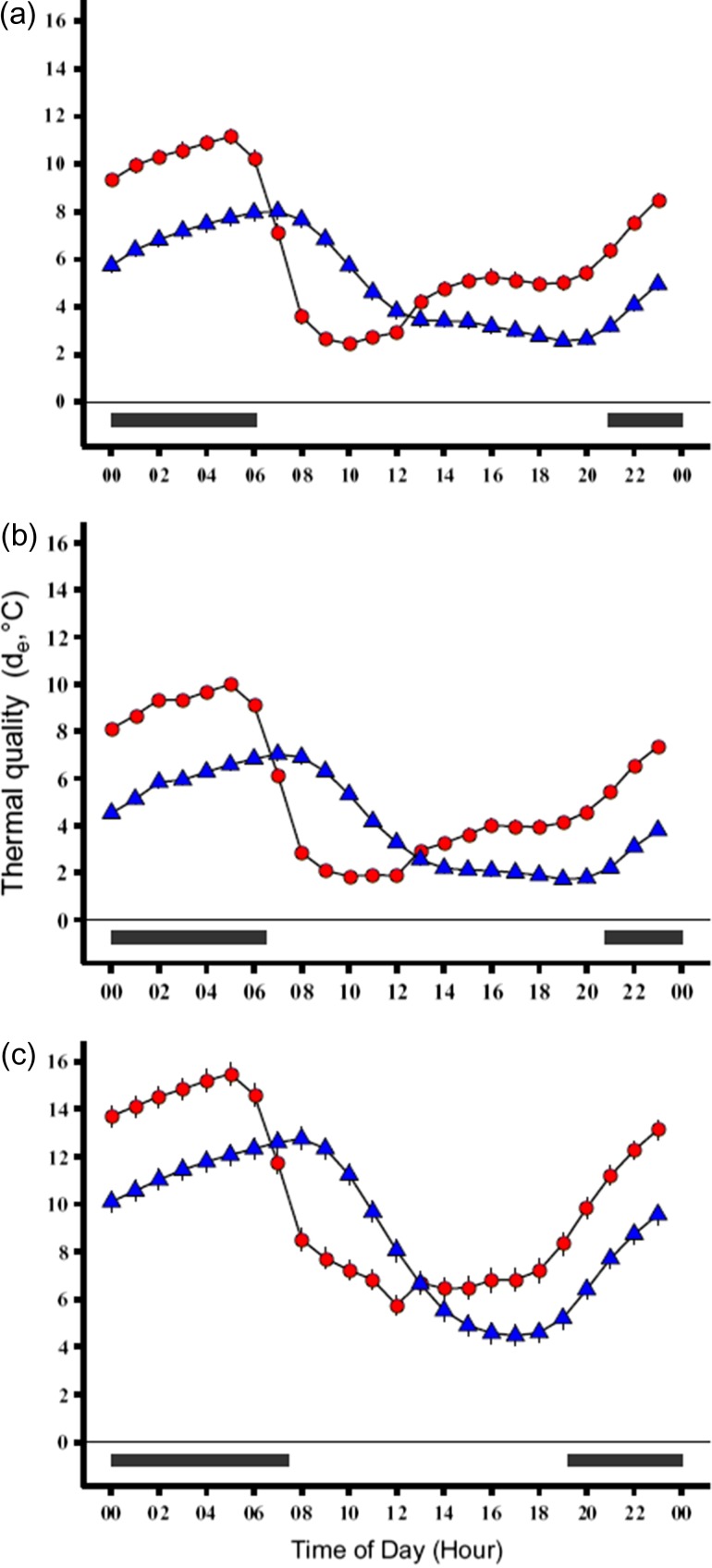
Thermal accuracy (*d*_b_ = │*T*_set_−*T*_b_│) plots for the beginning (**a**), middle (**b**) and end (**c**) of the active season for box turtles in a representative day. Blue triangles represent values calculated using *T*_set-C_ and the red circles represent values calculated using the *T*_set-D_. Black bars at the bottom represent night and are based on the photoperiod on (a) May 30, (b) July 30 and (c) September 30. Thermal accuracy values are higher (i.e. less accurate) at night than during the day for each season and each calculation (constant or dynamic), and values calculated from *T*_set-D_ had higher amplitude compared to the values calculated from *T*_set-C_.

Thermal quality (*d*_e_) across a representative day had a cyclic pattern. During the night and evening, *d*_e_ values were farther from preferred temperatures (*T*_e_ had greater deviation from *T*_set_ range), and during the day *d*_e_ values were closer to preferred temperatures (less deviation of *T*_e_ from the *T*_set_ range) for both constant and dynamic *T*_set_ calculations (Fig. 2). Furthermore, the narrower range of the dynamic *T*_set_ resulted in farther deviation from preferred temperatures (larger amplitudes) for *d*_e_ compared to those calculated from the constant *T*_set_. Across seasons, patterns of thermal quality (*d*_e_) shifted downward (decrease in value, increase in quality) from the beginning to the middle of the active season, and shifted upward (increase in value, decrease in quality) from the middle to the end of the active season (Fig. 2).

### Seasonal body temperature and thermal accuracy

Body temperature (*χ*^2^ = 21.0, df = 2, *P* < 0.05), shell temperature (*χ*^2^ = 22.3, df = 2, *P* < 0.05), and thermal accuracy (*d*_b_), calculated from both constant (*χ*^2^ = 23.9, df = 2, *P* < 0.05) and dynamic (*χ*^2^ = 21.8, df = 2, *P* < 0.05) *T*_set_, each had statistically significant year and season interaction effects. For *T*_b_, *T*_shell_, and *d*_b-C_ and *d*_b-D_, bootstrapping showed that both years and seasons were statistically similar. However, the end of the 2014 active season was different from other years and seasons. In 2014, average internal body temperature (*T*_b_) had a slight decrease (~0.30°C) from the beginning to middle of the active season and a larger decrease (~4.0°C) from the middle to the end. In 2015, *T*_b_ increased from the beginning to the middle of the active season, and then decreased from the middle to the end of the active season back to temperatures similar to those at the beginning (Table 3). Box turtles had lower thermal accuracy (*d*_b_) values, according to both types of *T*_set_ calculations, in the beginning and middle compared to the end of the active season in 2014, and had lower *d*_b_ values in the beginning and end compared to the middle of the active season in 2015 (Table 3).

**Table 3:** Internal body temperature (*T*_b_), external shell temperature (*T*_shell_), and thermal accuracy (*d*_b-C_; *d*_b-D_) of box turtles during the beginning (May and June), middle (July and August), and end (September and October) of their active season in 2014 (*n* = 14) and 2015 (*n* = 12)

	Season	*T*_b_ (°C)	*T*_shell_ (°C)	*d*_b-C_ (°C)	*d*_b-D_ (°C)
2014	Beginning	20.82 ± 0.94	20.33 ± 0.95	5.17 ± 0.87	6.56 ± 0.88
	Middle	20.58 ± 0.01	20.03 ± 0.01	5.32 ± 0.02	6.69 ± 0.01
	End	15.63 ± 0.74	14.72 ± 0.74	9.93 ± 0.67	11.31 ± 0.69
2015	Beginning	19.93 ± 1.17	19.57 ± 1.20	5.97 ± 1.10	7.42 ± 1.11
	Middle	21.81 ± 0.01	21.43 ± 0.01	4.12 ± 0.02	5.54 ± 0.01
	End	19.79 ± 2.20	19.05 ± 2.20	6.10 ± 2.00	7.44 ± 2.05

Higher values of *d*_b_ indicate greater deviation from the *T*_set_ range (low thermal accuracy) and lower values indicate *T*_b_ closer to the *T*_set_ range (high thermal accuracy). In 2014, *T*_b_ and *T*_shell_ remained similar in the beginning and middle of the active season and decreased towards the end. In 2015, *T*_b_ and *T*_shell_ increased from beginning to middle then decreased to temperatures similar to the beginning in the end of the active season. Thermal accuracy during both years reflects similar patterns. Data are presented as weighted means ± standard error.

**Figure 3:**
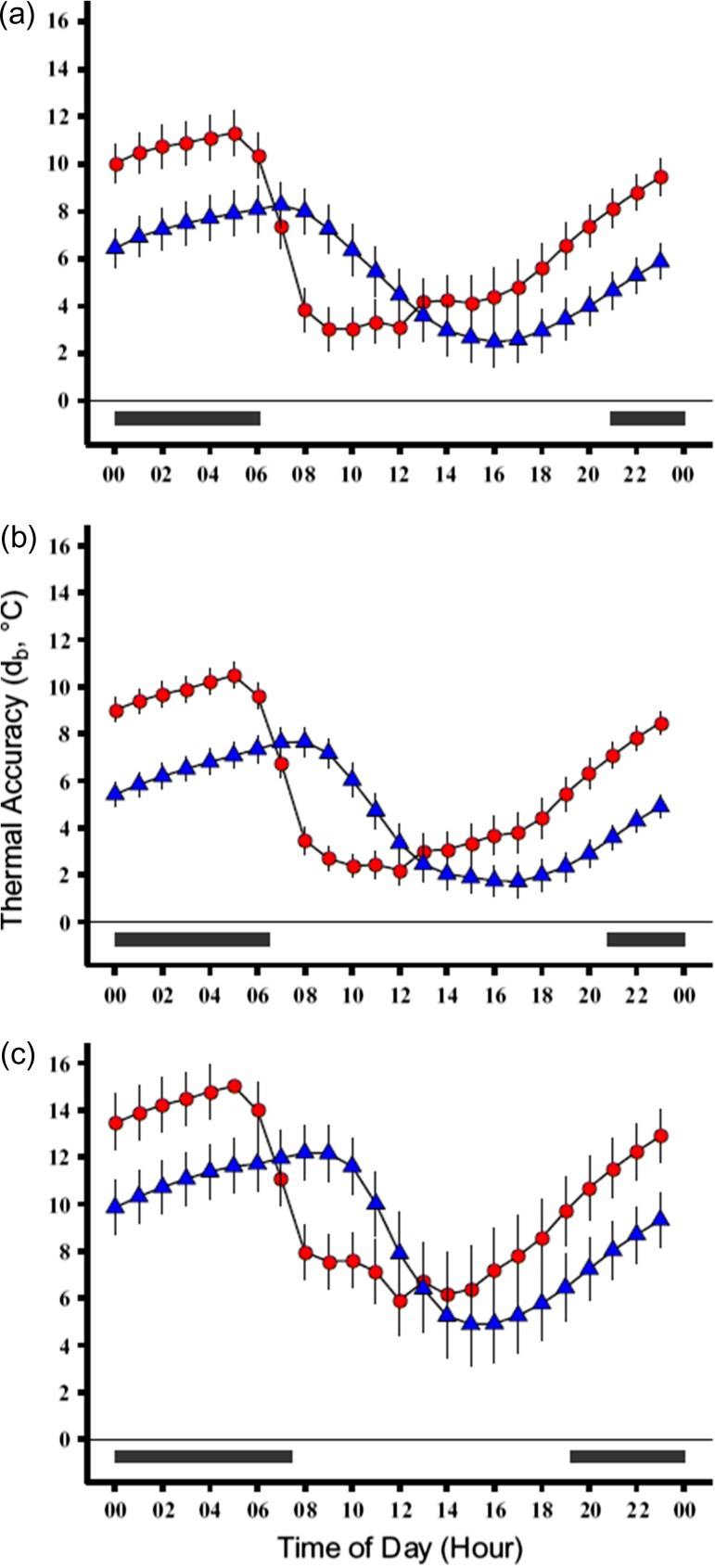
Thermal quality (*d*_e_ = │*T*_set_−*T*_e_│) plots for beginning (**a**), middle (**b**), and end (**c**) of the active season for box turtles in a representative day. Blue triangles represent values calculated using constant *T*_set_ and red circles represent those using dynamic *T*_set_ calculations. Black bars at the bottom represent night and are based on the photoperiod on (a) May 30, (b) July 30 and (c) September 30. Thermal quality values are higher (i.e. lower quality) at night than during the day for each season and each calculation (constant and dynamic). Values calculated from *T*_set-D_ had higher amplitude compared to the values calculated from *T*_set-C_.

Monitoring of body temperatures revealed patterns that were strikingly similar to those seen in the operative models. Calculations of thermal accuracy (*d*_b-C_, *d*_b-D_) in a representative day had a cyclic pattern wherein the largest deviation (higher values) from the preferred body temperature (*T*_set_) range was observed during the evening and night (Fig. 3). Thermal accuracy (*d*_b_) values decreased (lowering the deviation from *T*_set_) at dawn, and at dusk the *d*_b_ values increased (raising the deviation from *T*_set_). The narrower range of the dynamic *T*_set_ resulted in larger amplitudes of *d*_b_ in an average 24-h period (i.e. a representative day) compared to the amplitude produced from the constant *T*_set_ (Fig. 3). Patterns of thermal accuracy (*d*_b_) shifted downward (decrease in value, increase in accuracy) from the beginning to the middle of the active season, and shifted upward (increase in value, decrease in accuracy) from the middle to the end of the active season (Fig. 3).

### Effectiveness of thermoregulation and thermal exploitation

**Figure 4:**
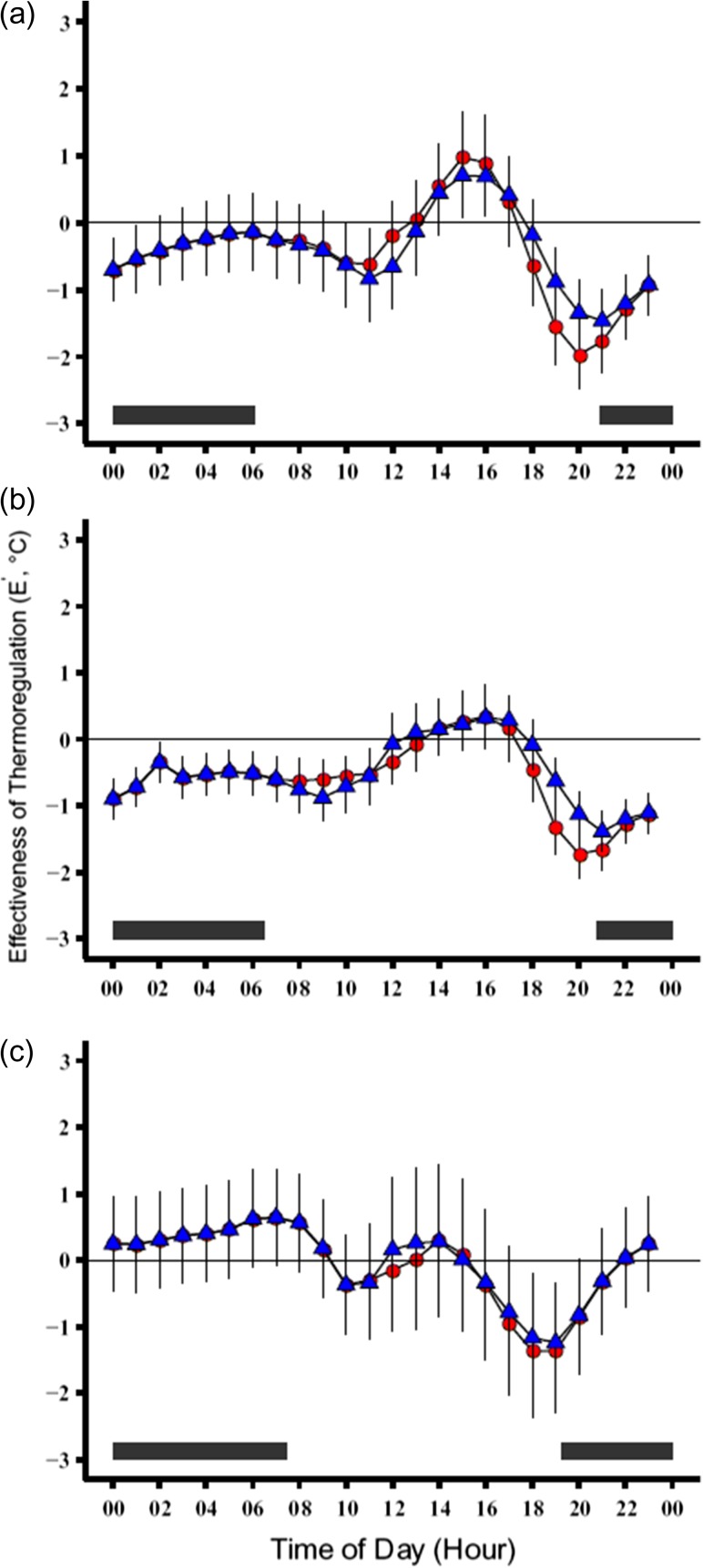
Effectiveness of thermoregulation (*E*′ = *d*_e_−*d*_b_) plots for the (**a**) beginning, (**b**) middle and (**c**) end of the active season for box turtles in a representative day. Blue triangles represent calculations from *T*_set-C_ and red circles represent *T*_set-D_. Black bars at the bottom represent night and are based on the photoperiod on (a) May 30, (b) July 30 and (c) September 30. Positive values indicate thermoregulation, values of zero are thermoconformity, and negative values indicate absence from thermally adequate habitat. Over a 24-h period box turtles remained near thermoconformity during each season. Additionally, there was a convergence of *E*′ values calculated from *T*_set-C_ and *T*_set-D_, indicating that both approaches revealed thermoconformity.

In 2014, the average effectiveness of thermoregulation (*E*′) value decreased towards thermoconformity (*E*′ = 0) from the beginning to the middle of the active season and then increased from the middle until the end of the active season (Table 4). In 2015, *E*′ values increased from the beginning to the end of the active season. In both years, the pattern for *E*′ was similar for both *T*_set-C_ (*E*′_-C_) and *T*_set-D_ (*E*′_-D_) calculations. When using all models from open and closed habitats, we found no significant interaction effect of season and year for *E*′_-C_ (*χ*^2^ = 1.8, df = 2, *P* > 0.05) or *E*′_-D_ (*χ*^2^ = 1.9, df = 2, *P* > 0.05). Given that turtles remained primarily in forested habitat, we recalculated the thermoregulatory indices using only the models from closed habitats to determine *E*′ using exclusively closed habitat conditions. We found that there were no significant interaction effects of season and year for *E*′_-C_ (*χ*^2^ = 0.8, df = 2, *P* > 0.05) or *E*′_-D_ (*χ*^2^ = 2.0, df = 2, *P* > 0.05). We did find significant main effects of year (*E*′_-C_, *χ*^2^ = 8.4, df = 1, *P* < 0.05; *E*′_-D_*χ*^2 ^= 11.5, df = 1, *P* < 0.05) and season (*E*′_-C_, *χ*^2^ = 12.3, df = 2, *P* < 0.05; *E*′_-D_, *χ*^2^ = 16.0, df = 2, *P* < 0.05) independently. Both effectiveness of thermoregulation calculations (*E*′_-C_, *E*′_-D_) using all models showed that turtles remained near thermoconformity across the entire active season (Fig. 4). However in a representative day, there were both positive (thermoregulation) and negative (presence in thermally unfavourable habitats) values calculated. During the beginning and middle of their active season, box turtles did not use thermally adequate habitats for the first half of the day, used such habitats during the mid-day, and then did not use them again in the evening and night. However, during the fall, box turtles also used thermally favourable habitats during the night (Fig. 4).

**Table 4:** Effectiveness of thermoregulation (*E*′) during beginning, middle, and end of the active season for 2014 (*n* = 14) and 2015 (*n* = 12)

	Season	*E*′ ± SE (*T*_set-C_)	*E*′ ± SE (*T*_set-D_)
2014	Beginning	0.48 ± 0.11	0.44 ± 0.12
	Middle	0.15 ± 0.13	0.04 ± 0.16
	End	1.35 ± 0.22	1.16 ± 0.24
2015	Beginning	−1.11 ± 0.13	−1.16 ± 0.17
	Middle	−0.59 ± 0.10	−0.67 ± 0.11
	End	0.91 ± 0.09	0.86 ± 0.08

Calculated *E*′ values in 2014 remained slightly above zero and in 2015 the beginning and middle of the active season were slightly below zero for both dynamic *T*_set_ range and constant *T*_set_ range. There were significant effects of season, and year, but not the interaction of season and year. Data are presented as weighted means ± standard error.

Micro-climate exploitation analysis showed that while box turtles frequently selected or experienced microclimates outside of their thermal preference, their internal body temperature was nonetheless sometimes within their preferred range. Using the constant thermal preference (*T*_set-C_) calculation, 2.3% of the hourly mean *T*_shell_ values were above the *T*_set-C_, 7.6% were inside the *T*_set-C_, and 89.8% were below the *T*_set-C_. Within each subset, internal body temperatures were occasionally inside *T*_set_ and, not surprisingly, this was most commonly seen when external temperatures were also within *T*_set_. For the times in which *T*_shell_ was above *T*_set-C_, internal body temperature (*T*_b_) was within *T*_set-C_ for 37.4% of those hours. Most occurrences were between 12:00 and 14:00 h, and there were no occurrences of Tb within *T*_set_ before 08:00 h or after 17 00 h. For the times in which *T*_shell_ was inside *T*_set-C_, *T*_b_ was inside *T*_set-C_ for 84.1% of those hours. Most occurrences were between 11:00 and 19 00 h, and there were no occurrences before 09:00 h or after 20:00 h. For the times in which *T*_shell_ was below *T*_set-C_, *T*_b_ occurred inside *T*_set-C_ for 1.0% of those mean hours. Most occurrences were between 17:00 and 19:00 h, and *T*_b_ was never within *T*_set_ before 14:00 h or after 21:00 h. For the dynamic calculations, 6.4% of the hourly mean *T*_shell_ values were above *T*_set-D_, 3.8% of the hourly values were inside the *T*_set-D_, and 89.8% were below the *T*_set-D_. Of the *T*_shell_ hours above *T*_set-D_, *T*_b_ occurred inside *T*_set-D_ for 18.6% of those hours. Most occurrences were between 11:00 and 14:00 h, with no occurrences before 08:00 h or after 18:00 h. Of the *T*_shell_ hours inside *T*_set-D_, *T*_b_ also occurred within *T*_set-D_ in 67.5% of those hours. Most occurrences were between 08:00 and 15:00 h, with no occurrences before 08:00 h or after 19:00 h. Of the *T*_shell_ hours below *T*_set-D_, *T*_b_ occurred inside *T*_set-D_ 0.6% of those hours. The highest occurrences were between 15:00 and 17:00 h, with no occurrences before 08:00 h or after 19:00 h. For all measures, box turtles seldom achieve their preferred body temperature when field conditions are not also inside *T*_set_.

### Effects of temperature on daily movement

**Figure 5:**
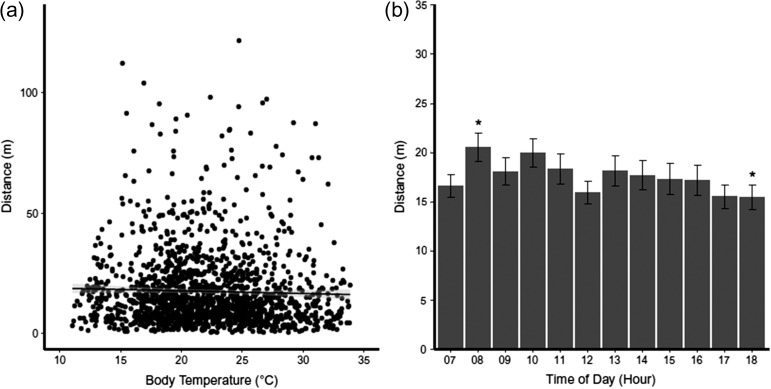
Distance moved plotted against body temperature (**a**) and time of day (**b**). In 2015, a total of 1666 GPS coordinates were obtained from 12 turtles. Body temperature and distance moved (a) between GPS points had no correlation. Time of day and mean (±standard error) hourly distance moved (b) was similar throughout the day but a significant difference between 08:00 and 18:00 h as indicated by the asterisk above each bar.

We found no correlation between the internal body temperature of box turtles and the distances travelled per hour (*r*^2^ = 0.0004, *F*_11,1534_ = 1.608, *P* = 0.20, Fig. 5a), showing that the extent of movement in the field does not depend on temperature. We did find a significant effect of time of day on distance moved (*F*_11,1534_ = 1.874, *P* < 0.05, Fig. 5b). However, pairwise comparisons showed no difference between the total distance moved in any two hours except that the distance moved after 18:00 h was 28.5% (5.1 m) less than that moved after 08:00 h.

## Discussion

Box turtles in Ohio are near the northwestern edge of their distribution (see review Dodd, 2001). Frequently, species at the extreme limits of their distributions are exposed to sub-optimal environmental conditions that can impact individual performance and in turn the demography and ecology of the species (Tuttle and Gregory, 2012). The goal of our study was to assess the importance of thermoregulatory behaviour on habitat selection and movement of box turtles, and to determine the extent to which the thermal quality of habitats influences box turtle habitat selection and movement ecology. We found (1) that habitat selected in this landscape did not reflect preferred body temperatures, (2) that the thermoregulatory strategy used by box turtles was close to thermoconformity, and (3) that movement of free-ranging turtles was not related to body temperature.

Our assessment of thermoregulation spanned the dynamic variation of free-living turtles in their native habitats. To reflect the potential that interactions with the environment may vary across the course of a given day, a novel aspect of our study was to incorporate time into the determination of thermal preference. Previous work on reptilian thermoregulation has determined the preferred temperatures as a constant value across a given day (e.g. Hertz *et al.*, 1993; Blouin-Demers and Weatherhead, 2001; Gvoždík, 2002; Rowe and Dalgarn, 2010). Because the optimal temperatures for locomotion, digestion and other physiological functions are not necessarily identical (see Angilletta, 2009) and because there may be different priorities among these functions across a 24-h period, we also determined the preferred temperature as an hourly function and compared the resultant indices of thermoregulation between these constant and dynamic conditions. Incorporating time of day into the preferred body temperature range revealed a strong diurnal fluctuation in the *T*_set_ range across a representative day, albeit these data were collected only in males. Preference for warmer temperatures at night may be driven by the need to maintain digestive function or may reflect attempts to buffer night-time heat loss and thus mitigate requirements for active thermoregulation in the morning. This interpretation fits the observed thermoconformity of box turtles during active periods, as warmer night-time refuges may make conformity less challenging in subsequent days. In either case, these data fit the observation that box turtles commonly excavate forms (shallow depressions in the soil) at night (Stickel, 1950) and thus likely reduce heat loss. Although, these are plausible explanations for our data, more detailed field studies will be required to test them. Similarly, it remains unknown if other species of reptiles exhibit similar diel patterns in body temperature selection.

Turtles were most commonly found in the closed, forested habitat, although these habitats had the least optimal temperatures. Nonetheless, habitat selected by box turtles in this study were similar to those selected in other studies of box turtles throughout their native range (see review Dodd, 2001). Peak temperatures measured in the operative models were higher in open than closed habitats and were frequently above 35°C in the middle of the active season. Above 35°C, box turtles engage mechanisms to lower their body temperature in order to avoid their upper lethal limit (43°C; Sturbaum, 1981). In a study of box turtle habitat quality, Reagan (1974) also noted that forests had higher humidity and greater levels of ground moisture than in the surrounding open areas. Therefore, forest habitats might not be optimal for thermal function, but these habitats might facilitate avoidance of excessive temperatures, reduce the risk of desiccation, and provide increased access to other resources. These advantages might over-ride thermal considerations. It is also possible that forest habitats in warmer parts of the box turtles’ range provide adequate thermal conditions, in which case the thermoregulatory behaviour that we observed in this region could be determined by the box turtles’ evolutionary history.

Regardless of whether we used the constant or dynamic measure of preferred temperature, we found that box turtles in Ohio were thermoconformers. Effectiveness of thermoregulation (*E*′) was near zero (thermoconformity) in nearly all cases, including analysis with all operative models or with only those placed in the closed, forest habitats, although *E*′ did show positive values in the afternoons in this latter case. Nonetheless, the values for *E*′ never exceeded values of 1.5°C in a representative day and fluctuated around zero (positive and negative values), indicating minimal divergence of body temperature from that of the models. In active thermoregulators, such as the atlas gecko (*Quedenfeldtia trachyblepharus*), values for *E*′ range from 2.1 to 12.5°C throughout a given active season (Bouazza *et al.*, 2016). Other active thermoregulators near their northern population limit, such as massasauga rattlesnakes (*Sistrurus catenatus*), have *E*′ that vary from 2.5 to 4.4°C (Harvey and Weatherhead, 2010). The turtles were both found in thermally sub-optimal habitats and apparently not using behavioural mechanisms to achieve selected body temperature. This suggests that thermal quality of habitats may not be of primary importance for these animals.

Although values for *E*′ did not depend on the method for determining preferred temperatures, we found that calculated values of thermal accuracy (*d*_b_) and thermal quality (*d*_e_) using the constant and dynamic *T*_set_ ranges produced different values, and these suggested different interpretations. Average values for both *d*_b_ and *d*_e_ calculated using the dynamic *T*_set_ were higher than those using the constant *T*_set_, reflecting the narrower range of preferred temperatures obtained using the hourly values and suggesting that turtles are even less able to achieve preferred temperatures (*d*_b_) or to find high quality thermal environments (*d*_e_). Calculations of hourly patterns in thermal accuracy and quality differed as much as 5°C when comparing the values obtained using the constant *T*_set_ or dynamic *T*_set_ values. The daily pattern also differed qualitatively, as the change between consecutive hourly averages was more gradual for the constant *T*_set_ than the dynamic *T*_set_. Furthermore, the amplitude of the daily cycle was greater when calculated with the dynamic *T*_set_ reflecting the failure to find adequate thermal habitats or to attain preferred temperatures at night. These data suggest that the dynamic *T*_set_ may be able to detect subtle changes in thermoregulatory behaviour that would otherwise not be observed with the constant *T*_set_ range. These data also reinforce the observation that thermal quality of selected habitats is considerably divergent from their preferred temperatures. Regardless of the metric used, we found that box turtles tended to be thermoconformers and were found in habitat that was thermally unfavourable. These findings extended across both years and in every season, although care should be taken in the interpretation at finer scales as the sample size was sometimes quite low. These findings also have implications for our understanding of the factors driving habitat choice, and responses to changing thermal conditions or human alterations of available landscapes as laboratory assumptions of thermal preferences clearly do not match field behaviours.

Given the literature on the temperature dependence of ectotherm locomotion (Navas *et al.*, 1999; Elnitsky and Claussen, 2006; Lailvaux and Irschick, 2007; Schaeffer and Lindstedt, 2013), we anticipated that box turtles would move greater distances when warmer and that the limited availability to optimal temperatures would strongly impact movements. Previous work on box turtles supported this expectation as Adams *et al.* (1989) found that **T*. carolina* had their highest locomotor performance with body temperatures between 24°C and 32°C. However, we found no correlation between box turtle movement and body temperature. Although we found a significant difference between movement and time of day, the lack of a general pattern observed suggests that this is not likely of biological significance. Box turtles have been recorded moving distances of 200 m/day (Claussen *et al.*, 1997), and we have observed turtles moving greater than 400 m in a given day (pers. obs., Parlin). These turtles appear equally capable of fulfilling their daily movement requirements across the full range of body temperatures experienced. However, longer distance movements, which we did not observe in this study but that box turtles occasionally undertake, may only be possible at higher body temperatures. Moreover, Iglay *et al.* (2007) found that in fragmented landscapes, box turtles moved less in isolated areas and more in continuous areas, showing that isolation of forest patches can impact movement and thus habitat selection, potentially limiting population viability.

## Conclusions

Although suitable thermal habitat is available to box turtles in Ohio, detailed thermoregulatory analysis clearly indicates that they do not use that resource, instead remaining in the cooler forest canopy. As Ohio is near the northern limit of box turtles, the length of their active season for growth and reproduction is likely limited due to sub-optimal conditions (Tuttle and Gregory, 2012). Although box turtles are thermoconforming in the available sub-optimal thermal conditions, their movement patterns are surprisingly unaffected by body temperature. This suggests that they are capable of accomplishing essential activities in spite of this ‘thermal marginalization.’ Although box turtles possess some capability to tolerate sub-zero temperatures and internal ice formation (Costanzo and Claussen, 1990), low-temperature induced mortality occurs during overwintering and may be more important for limiting this population than the cool summer temperatures. As thermoconformers are generally more likely to be impacted by climate warming, box turtles and potentially other similarly behaving ectotherms may benefit from higher temperatures. However, this prospect of change is made more complex by the challenges posed by forest fragmentation and agricultural impacts given box turtles’ reliance on intact, continuous forest and avoidance of more open habitats. Finally, multiple measures of animal–habitat interaction all indicate that box turtles live in thermally sub-optimal habitats. These data strongly suggest that models of habitat requirement must include data on organismal physiology to avoid errors.
